# Supplementary material for: Different Extraction Procedures Revealed the Anti-Proliferation Activity from Vegetable Semi-Purified Sources on Breast Cancer Cell Lines
Source: Antioxidants (Basel). 2023 Jun 9;12(6):1242. doi: 10.3390/antiox12061242 (PMC10295039; doi:10.3390/antiox12061242)
Supplement: Supplementary file 1 [file antioxidants-12-01242-s001.zip › antioxidants-2390228-supplementary.pdf]

Supplementary data

Extracts purification. The extracts purification was performed as described in Materials and Methods section. The chromatograms, obtained by the purification of each vegetable extract used in this study, are reported below.

Aloe vera

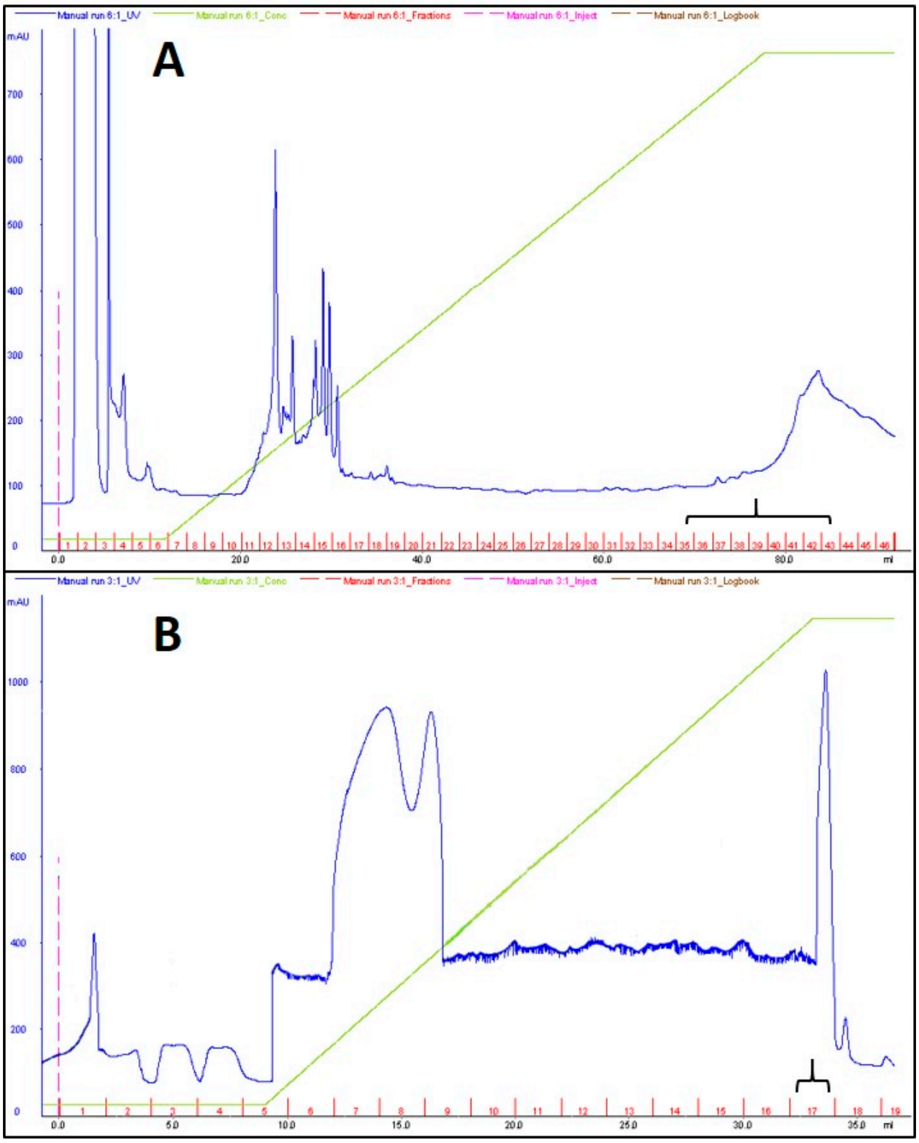

Figure S1. *Aloe vera* leaves extract purification. (**A**) Chromatogram related to the crude extract purification on a Source 5RPC ST 4.6/150 column (GE Healthcare, USA; volume column 2.5 ml, flow rate 0.5 ml/min). The column was equilibrated with Acetonitrile 10% (buffer A), washed with 10 ml of buffer A and eluted with 60 ml of buffer B (acetonitrile 100%), using a linear gradient 0-100%B; The fractions from 35 to 43, showing anti-proliferative activity, were pooled and dried. (**B**) After dissolving in 2 ml of methanol the pool, it was loaded onto a µRPC C2/C18 ST 4.6/100 column (GE Healthcare, USA; volume column 1.5 ml, flow rate 0.4 ml/min), previously equilibrated with buffer A. After washing (10 ml buffer A), sample was eluted with 25 ml of buffer B. The fraction 17 exhibited anti-proliferative activity.

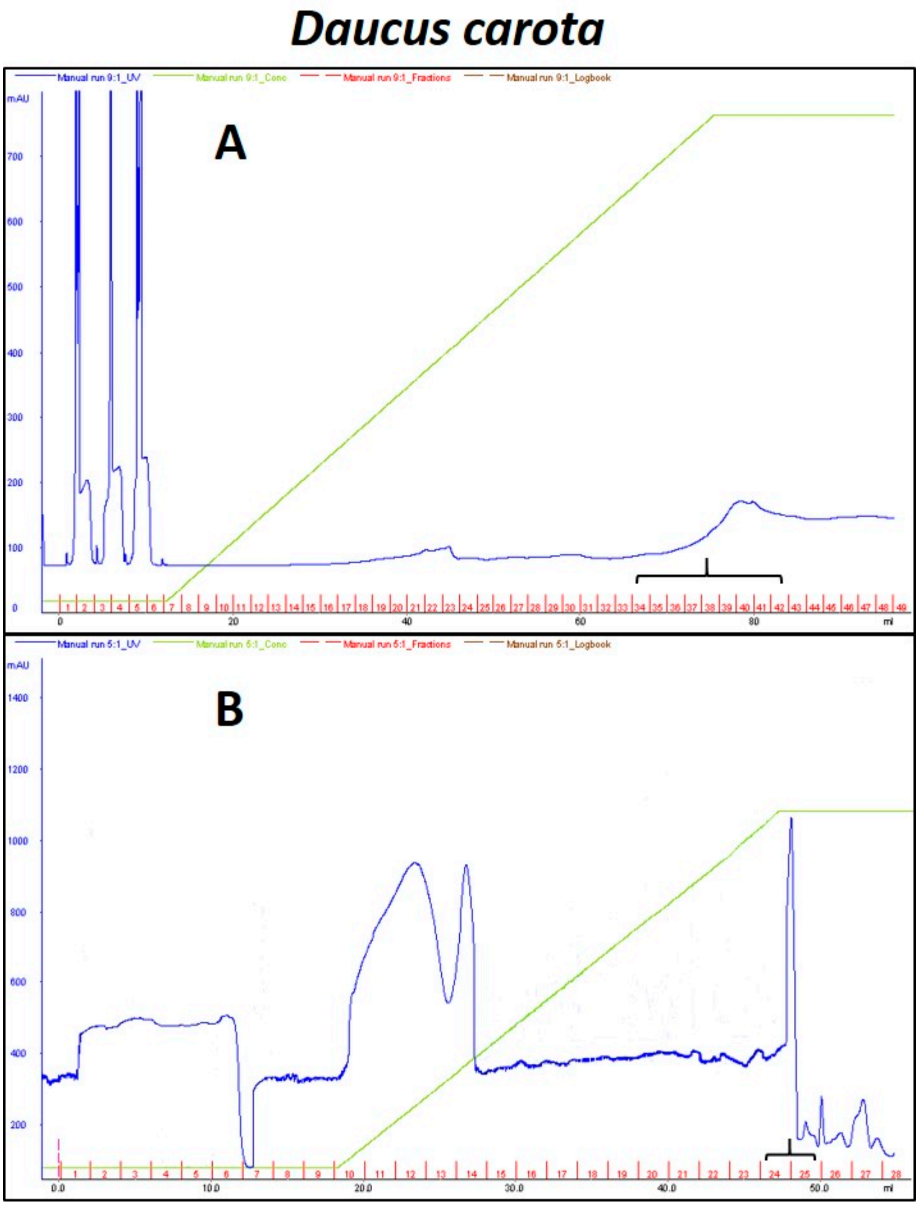

Figure S2. ***Daucus carota*** **extract purification.** (**A**) Chromatogram related to the crude extract purification on a Source 5RPC ST 4.6/150 column (GE Healthcare, USA; volume column 2.5 ml, flow rate 0.5 ml/min). The column was equilibrated with Acetonitrile 10% (buffer A), washed with 10 ml of buffer A and eluted with 60 ml of buffer B (acetonitrile 100%), using a linear gradient 0-100%B; The fractions from 34 to 42 showing anti-proliferative activity were pooled and dried. (**B**) After dissolving in 2 ml of methanol the pool was loaded onto a µRPC C2/C18 ST 4.6/100 column (GE Healthcare, USA; volume column 1.5 ml, flow rate 0.4 ml/min), equilibrated with buffer A. After washing (20 ml buffer A) sample was eluted with 25 ml of buffer B. The fractions 24 and 25 showed anti-proliferative activity.

Calendula officinalis

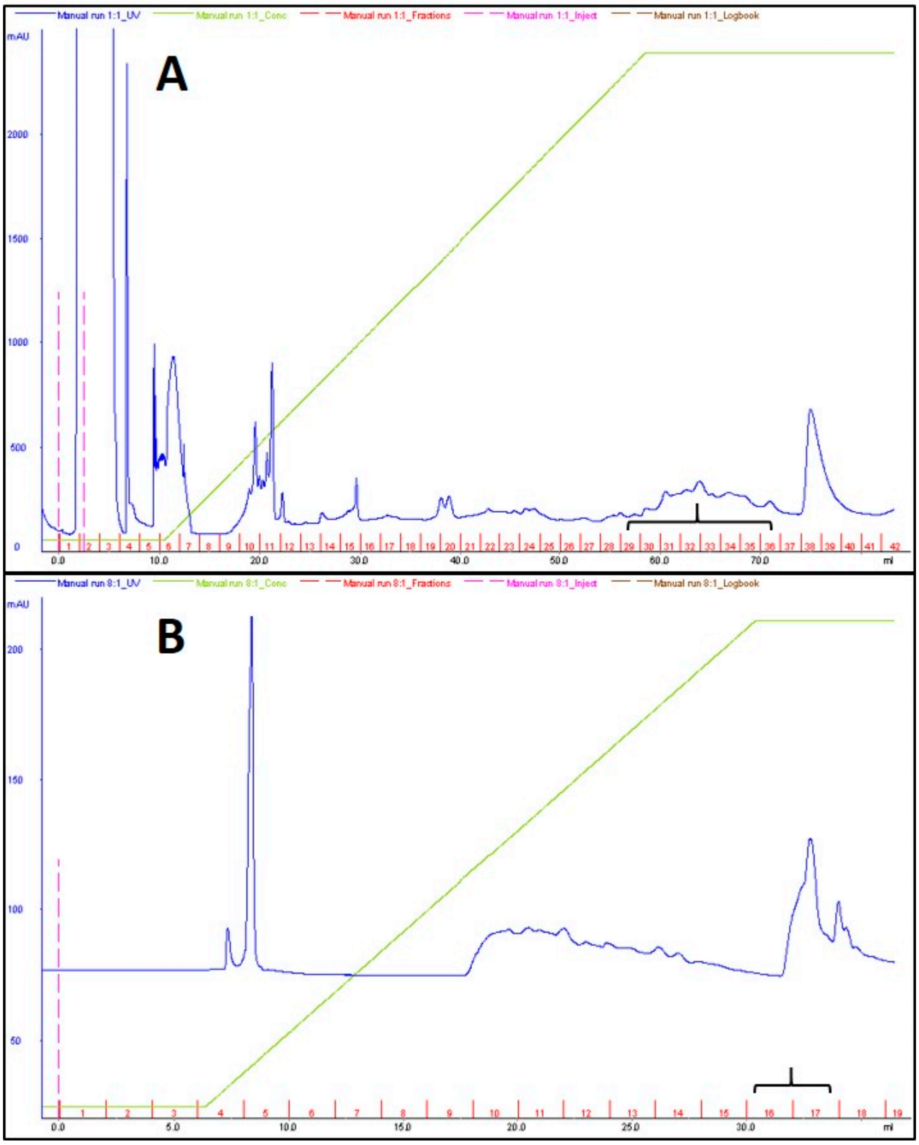

Figure S3. ***Calendula officinalis* flowers extract purification.** (**A**) Chromatogram related to the crude extract purification on a Source 5RPC ST 4.6/150 column (GE Healthcare, USA; volume column 2.5 ml, flow rate 0.5 ml/min). The column was equilibrated with Acetonitrile 10% (buffer A), washed with 10 ml of buffer A and eluted with 60 ml of buffer B (acetonitrile 100%), using a linear gradient 0-100%B; The fractions from 29 to 36 showing anti-proliferative activity were pooled and dried. (**B**) After dissolving in 2 ml of methanol the pool was loaded onto a µRPC C2/C18 ST 4.6/100 column (GE Healthcare, USA; volume column 1.5 ml, flow rate 0.4 ml/min), equilibrated with buffer A. After washing (5 ml buffer A) sample was eluted with 25 ml of buffer B. The fractions 16 and 17 showed anti-proliferative activity.

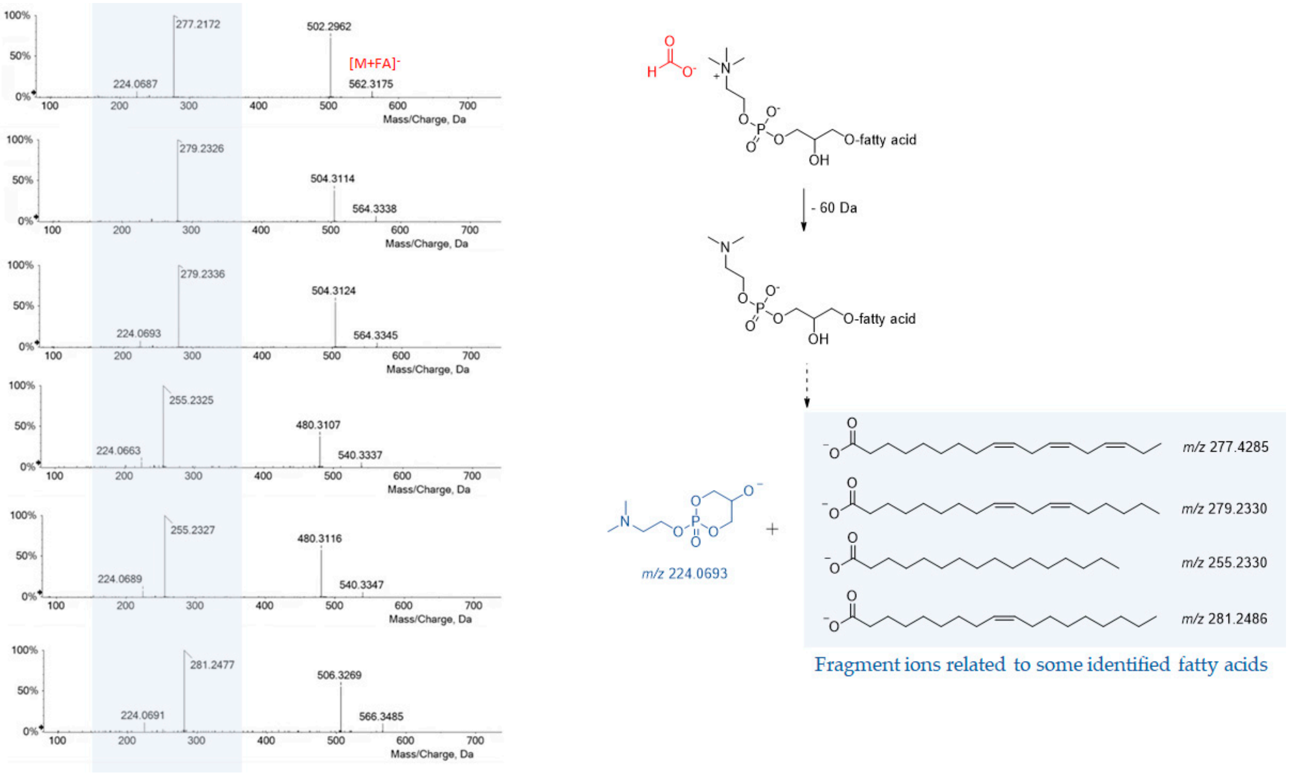

Figure S4. HR-MS/MS spectra of glycerophosphocholine derivatives. Some ions pivotal for identification are highlighted (theoretical *m/z* values are associated to each structure).

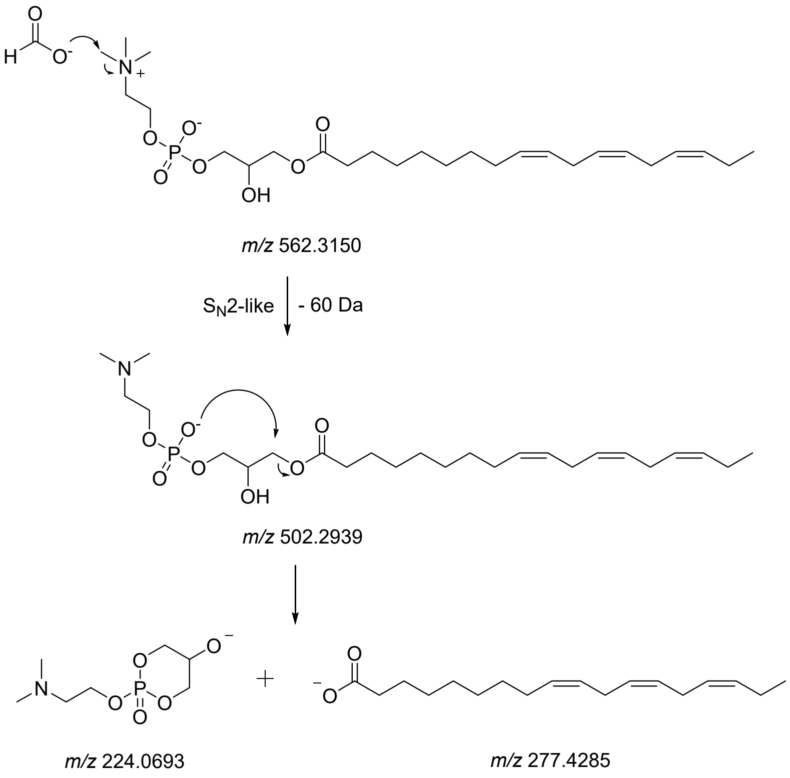

Figure S5. Insights into the fragmentation pathway of GPC(18:3), based on HR-MS/MS spectrum.
